# Spatial Analysis of Schistosomiasis in Hubei Province, China: A GIS-Based Analysis of Schistosomiasis from 2009 to 2013

**DOI:** 10.1371/journal.pone.0118362

**Published:** 2015-04-07

**Authors:** Yan-Yan Chen, Xi-Bao Huang, Ying Xiao, Yong Jiang, Xiao-wei Shan, Juan Zhang, Shun-Xiang Cai, Jian-Bing Liu

**Affiliations:** Hubei Center for Disease Control and Prevention, Wuhan, China; Centro de Pesquisa Rene Rachou/Fundação Oswaldo Cruz (Fiocruz-Minas), BRAZIL

## Abstract

**Background:**

Schistosomiasis remains a major public health problem in China. The major endemic areas are located in the lake and marshland regions of southern China, particularly in areas along the middle and low reach of the Yangtze River. Spatial analytical techniques are often used in epidemiology to identify spatial clusters in disease regions. This study assesses the spatial distribution of schistosomiasis and explores high-risk regions in Hubei Province, China to provide guidance on schistosomiasis control in marshland regions.

**Methods:**

In this study, spatial autocorrelation methodologies, including global Moran’s *I* and local Getis–Ord statistics, were utilized to describe and map spatial clusters and areas where human *Schistosoma japonicum* infection is prevalent at the county level in Hubei province. In addition, linear logistic regression model was used to determine the characteristics of spatial autocorrelation with time.

**Results:**

The infection rates of *S. japonicum* decreased from 2009 to 2013. The global autocorrelation analysis results on the infection rate of S. japonicum for five years showed statistical significance (Moran’s *I* > 0, *P* < 0.01), which suggested that spatial clusters were present in the distribution of *S. japonicum* infection from 2009 to 2013. Local autocorrelation analysis results showed that the number of highly aggregated areas ranged from eight to eleven within the five-year analysis period. The highly aggregated areas were mainly distributed in eight counties.

**Conclusions:**

The spatial distribution of human *S. japonicum* infections did not exhibit a temporal change at the county level in Hubei Province. The risk factors that influence human *S. japonicum* transmission may not have changed after achieving the national criterion of infection control. The findings indicated that spatial–temporal surveillance of *S. japonicum *transmission plays a significant role on schistosomiasis control. Timely and integrated prevention should be continued, especially in the Yangtze River Basin of Jianghan Plain area.

## Introduction

Schistosomiasis is a parasitic disease caused by trematode flukes of the genus *Schistosoma*. By conservative estimates, at least 230 million people are infected with *Schistosoma* in 76 countries and territories in the world [1,2]. The main species of *Schistosoma* that infect human beings are *Schistosoma haematobium*, *Schistosoma mansoni*, and *Schistosoma japonicum*. In China, the main species is *S*. *japonicum*. Documented evidence indicates that *S*. *japonicum* has been endemic for a long time in China [3]. *S*. *japonicum* eggs were identified in a male corpse dating back to the Western Han dynasty some 2100 years ago that was exhumed in Jianglin Hsien, Hubei Province in 1975 [4]. *S*. *japonicum* eggs were also found in a female corpse buried at about the same time in Hunan Province [5].

In China, schistosomiasis is mainly endemic in lake and marshland areas (Hubei, Hunan, Jiangxi, Anhui, and Jiangsu provinces) and in hilly and mountainous regions (Sichuan and Yunnan provinces) [6]. The Chinese government has given high priority to the control of schistosomiasis in the 1950s. Since 2005, an integrated strategy that emphasizes humans and cattle as the main infection source control has been carried out in the schistosomiasis-endemic areas of China. Significant achievements on schistosomiasis control have been attained in the past 50 years [7]. The third nationwide schistosomiasis sampling survey indicated that the number of schistosomiasis patients decreased by 55.7%, from 1,638,103 cases in 1989 to 726,112 cases in 2004 [8].

Hubei Province is a highly schistosomiasis-endemic area in China that is located in the middle reaches of the Yangtze River. Affected by flood along the upper reaches of the Yangtze River, the marshlands along the Yangtze River operate in a “land in winter, water in summer” cycle; they are ideal breeding sites for *Oncomelania hupensis* snails [9,10]. *O*. *hupensis* is the unique intermediate host of *S*. *japonicum*, which has a key function during the transmission of schistosomiasis. By the end of 2004, Hubei Province had 5,499 schistosomiasis-endemic villages and 292,059 cases of chronic schistosomiasis; the prevalence of schistosomiasis in humans and bovines was 3.9% and 6.2% at the province level, respectively [11]. A document titled “Mid- and long-term plan on prevention and control of schistosomiasis in Hubei Province (2005–2015)” was formulated to reduce the transmission of *S*. *japonicum* in Hubei Province. Since 2005, the whole province has carried out an integrated control strategy aimed at reducing the roles of humans and cattle, which includes human chemotherapy, health education/promotion, measures of improving water supply and sanitation, mollusciciding, and bovine chemotherapy [12]. By the end of 2008, the prevalence of human *S*. *japonicum* infection decreased to below 5% at the village level and no acute schistosomiasis case has been reported in Hubei Province. Since then, Hubei province has achieved the national criterion of infection control, which defined the prevalence of human *S*. *japonicum* infection decreased to below 5% at the village level. However, snails may diffuse and schistosomiasis prevalence may rebound because the environment has not fundamentally changed, coupled with the effect of floods and other natural factors. Therefore, the integrated control program has been continuously carried out in Hubei Province since 2008.

The use of Geographic Information System (GIS) helps in elucidating the actual distribution of schistosomiasis; it is an effective tool for planning and monitoring the disease at a local level [13,14]. A number of studies have reported that schistosomiasis prevalence showed spatial and/or temporal patterns [15–17]. However, few studies have analyzed the space–time changes of schistosomiasis infection at the county level in Hubei Province, especially after the whole province reached the national criterion of infection control.

In this study, the spatio–temporal distribution and variation of schistosomiasis in Hubei Province from 2009 to 2013 were investigated to evaluate the progress of the integrated control strategy after achieving the national criterion of infection control. The provincial database on schistosomiasis was used to disclose the spatial cluster using global and local spatial autocorrelation analyses.

## Materials and Methods

### Study area

**Fig 1:**
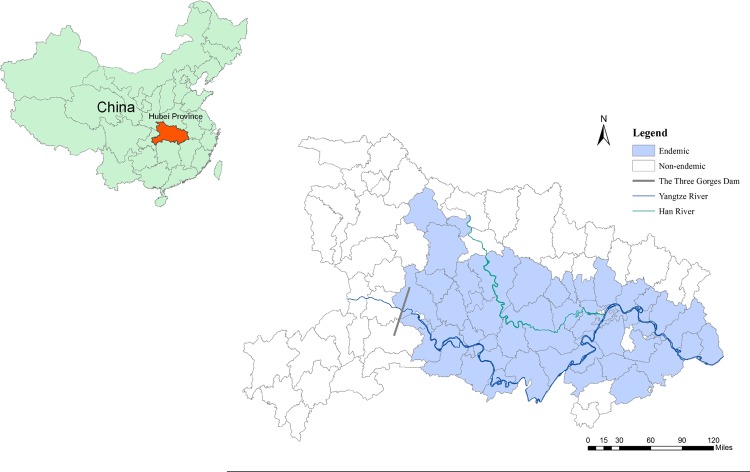
Location of the study areas in Hubei Province, China.

The study focused on Hubei Province (29°05′–33°20′ N, 108°21′–116°07′ E), which is located in the middle reaches of the Yangtze River in central China (Fig. 1). The region has an area of 185,900 km^2^ and a population of 57.8 million. The landscape is divided into 17 cities and 103 counties.

Schistosomiasis is mainly endemic in 13 cities and 63 counties, which are mainly distributed in the Yangtze River Basin and in the Jianghan Plain [18,19]. There were about 9.8 million people who were at risk of infection with schistosomiasis.

### Data collection

**Schistosomiasis endemiologic data.** Data on human *S*. *japonicum* infections were obtained from the annual surveillance that covered all the schistosomiasis-endemic areas in Hubei Province from 2009 to 2013. In each of the study villages, more than 90% of the residents aged between 6 and 65 years old were screened annually using indirect hemagglutination assay (IHA) [20,21] in October and November. Stool samples were then collected from IHA-positive individuals to conduct miracidium-hatching test [22–24]. Residents that were positive for both IHA and stool test were defined as infected, and the prevalence of human *S*. *japonicum* was determined in autumn.

**Map data.** The map used for the administrative division of Hubei Province was obtained using ArcGIS10.1 software (ESRI, Redlands, CA, USA). GCS_Krasovsky_1940 and Krasovsky_1940_Albers were used to determine the geographic coordinate system and projection coordinate system, respectively.

### Construction of spatial database

Each county was designated a code in the map data of Hubei Province. First, the code for each county in the schistosomiasis database was set up according to the code in the map data. Then, the schistosomiasis data were coupled with the geographic database by matching with the code in ArcGIS10.1. Hence, the schistosomiasis spatial database of Hubei Province was established, and spatial analysis for schistosomiasis was conducted.

### Descriptive analysis

The spatial distribution of human *S*. *japonicum* infection at the county level of the whole province from 2009 to 2013 was shown as a visual description by ArcGIS10.1 based on the spatial database.

### Spatial cluster analysis

The spatial distribution of schistosomiasis was evaluated by spatial autocorrelation analysis at the county level to describe the correlation of disease distribution and the spatial distribution forms in the research areas [25]. The input field for spatial analysis was the prevalence of human *S*. *japonicum* infection per county.

### Global spatial autocorrelation analysis

Global spatial autocorrelation analysis was used to measure the correlation among neighboring observations to detect whether the pattern was clustered, dispersed, or random [26]. Moran’s *I* is mainly used to estimate the independence or correlation of neighboring counties. Moran’s *I* statistics is defined as follows:

$$I=\frac{n•\sum_{i=1}^{n}\sum_{j=1}^{n}w_{ij}\left(x_{i}-x\right)\left(x_{j}-x\right)}{\left(\sum_{i=1}^{n}\sum_{j=1}^{n}w_{ij}\right)•\sum_{i=1}^{n}{\left(x_{i}-x\right)}^{2}},i\neq j$$ 1

where *n* is the number of counties; *x* is the average prevalence in the counties; *X*_*i*_ and *X*_*j*_ are the prevalence in counties *i* and *j*, respectively; and *W*_*ij*_ is the spatial weight between counties *i* and *j*. Z test is usually used as a hypothesis test to state whether a spatial clustering exists [27]. Thus, Z scores greater than 1.96 or smaller than −1.96 indicate significant spatial autocorrelation at the 5% level.

Moran’s *I* statistics range between −1 to +1. Based on the null hypothesis of complete spatial randomness, a Moran’s *I* value near 0 indicates a lack of spatial pattern (values observed at one location do not depend on values observed at neighboring locations). Positive coefficients reflect neighboring areas with similarly large or small values, whereas negative coefficients reflect neighboring areas with large inverse values.

### Local spatial autocorrelation analysis

The overall clustering tendency of the disease risk in the study region was assessed by global spatial autocorrelation test, which only investigates the presence but not the exact location of the cluster(s). Therefore, local spatial autocorrelation analysis was conducted to test the statistical significance of local clusters and to detect the spatial extent of these clusters [28–30]. Getis–Ord Gi* (Gi*) was used to identify the individual members of the local clusters. The Gi* statistics is written as follows [31,32]:

$$G_{i}^{*}=\frac{\sum_{j=1}^{n}w_{i,j}x_{j}-x\sum_{j=1}^{n}w_{i,j}}{\sqrt{\frac{\sum_{j=1}^{n}x_{j}^{2}}{n}-x^{2}}\sqrt{\frac{\left[n\sum_{j=1}^{n}w_{i,j}^{2}-{\left(\sum_{j=1}^{n}w_{i,j}\right)}^{2}\right]}{n-1}}}$$ 2

where *x* is the average infection rate of *S*. *japonicum* in the counties; *X*_*j*_ is the prevalence on county *j*; and *W*_*ij*_ is the spatial weight that defines neighboring administrative districts *j* to *i*. The output of Gi* statistics can be calculated as a standard normal variant with an associated probability from the Z-score distribution [33]. Gi* > 0 and *Z* > 1.96 indicate that the study area is significantly clustered with a high value and is considered a “hotspot”, whereas Gi* < 0 and *Z* < −1.96 indicate that the study area is significantly clustered with a low value and is considered a “cold spot”. When Gi* > 0 and *Z* ≤ 1.96 or Gi* < 0 and *Z* ≥ −1.96, the study area is not clustered and the sites are distributed in random.

The global and local spatial autocorrelation analyses were all performed in the ArcGIS10.1 software.

### Ethical approval

The research was approved by the Ethics Review Committee of Hubei Provincial Center for Disease Control and Prevention, Wuhan, China. Written informed consents were obtained from all residents and from the parents or guardians of minors before participation in the study. The participants had the opportunity to withdraw from the study at any time. During the study period, all participants who tested positive for *S*. *japonicum* were treated with praziquantel (PZQ) (40 mg/kg), in accordance to the World Health Organization recommendation.

## Results

### Spatial distribution of *S*. *japonicum* in Hubei Province

**Fig 2:**
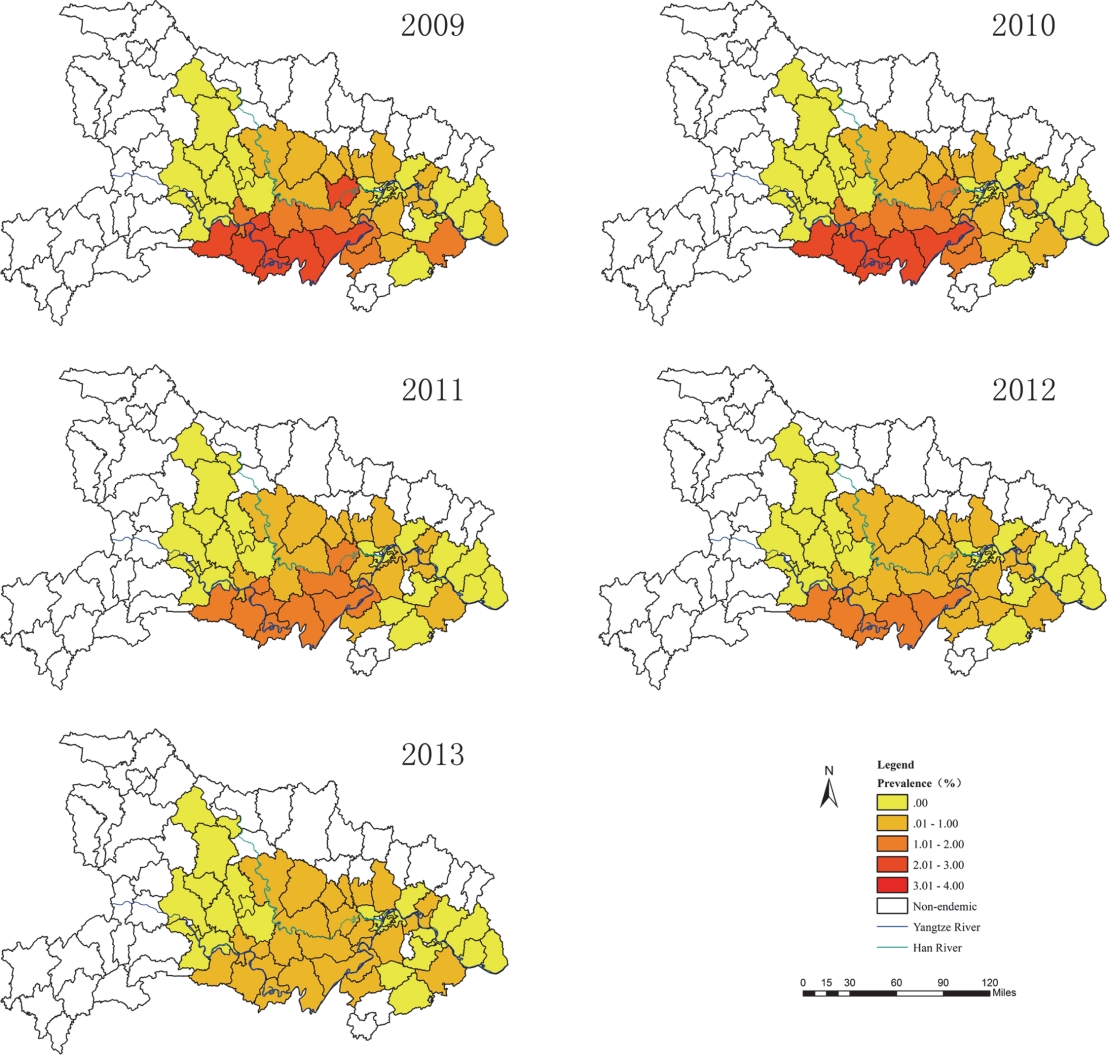
The distribution of human *S*. *japonicum* infection at the county level in Hubei Province from 2009 to 2013.

A total of 207,359 cases of *S*. *japonicum* patients in Hubei Province in 2009, which decreased to 120,990 cases in 2013 (Table 1), were studied. In the study years, the maximum prevalence of human *S*. *japonicum* infection at the county level ranged from 2.7 in 2009 to 0.7 in 2013, showing a declining trend (Table 2). In 2009, among the 63 total endemic counties in Hubei Province, nine counties were highly endemic (prevalence: 2%–3%, covering 14.3% of total land). In 2010, six counties were highly endemic (prevalence: 2%–3%, covering 9.5% of total land). The highest prevalence of human *S*. *japonicum* infection was 1%–2% in 2011 and 2012, with 11 and 5 counties (covering 17.5% and 7.9% of total land), respectively (Fig. 2). The prevalences of human *S*. *japonicum* infection at the county level were all reduced to less than 1% in 2013.

**Table 1:** The dynamic change in schistosomiasis patients in Hubei Province from 2009 to 2013*.

Year	Total	Minimum	*P*_25_	Median	*P*_75_	Maximum
**2009**	207359	0	1	31	2353	36612
**2010**	186948	0	1	91	2136	34749
**2011**	177424	0	4	102	1982	33069
**2012**	146484	0	1	81	1860	26353
**2013**	120990	0	2	66	1772	21590

*The columns represent the number of schistosomiasis patients.

**Table 2:** The dynamic change in the prevalence of human *S*. *japonicum* infection in Hubei Province from 2009 to 2013*.

Year	Minimum (%)	*P*_25_ (%)	Median (%)	*P*_75_ (%)	Maximum (%)
**2009**	0	0	0.0010	0.8500	2.7700
**2010**	0	0	0	0.6336	2.3400
**2011**	0	0	0	0.4700	1.6000
**2012**	0	0	0	0.3876	1.3500
**2013**	0	0	0	0.2800	0.7100

*The columns represent the prevalence of human *S*. *japonicum* infection.

### Spatial cluster of *S*. *japonicum* in Hubei Province

**Fig 3:**
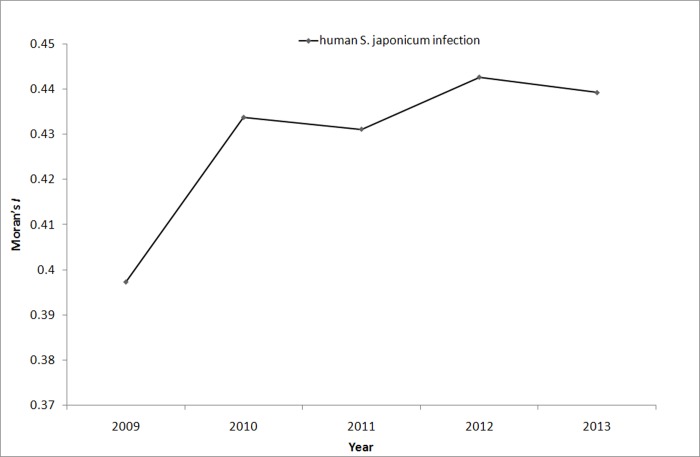
The dynamic change of Moran’s *I* value of human *S*. *japonicum* infection in Hubei Province from 2009 to 2013.

Global Moran’s *I* statistics was performed to determine the presence of global autocorrelation from 2009 to 2013. The results of the global autocorrelation statistics for human *S*. *japonicum* infection in each year are summarized in Table 3. The results demonstrated that high global spatial autocorrelation of *S*. *japonicum* was detected at the county level in Hubei Province from 2009 to 2013 (Moran’s *I* > 0.4, *P* < 0.01). This finding showed that counties with high *S*. *japonicum* infection gathered in other counties with high infection. The results of regression analysis of Moran’s *I* statistics and time value did not show any linear trend (*β* = 0.836, *t* = 2.642, *P* = 0.078), which meant that the spatial cluster of *S*. *japonicum* in Hubei Province did not exhibit a temporal change (Fig. 3).

**Table 3:** The global autocorrelation analysis of *S*. *japonicum* infection in humans.

Year	Moran’s *I*	Expected index	Variance	Z Score	*P*_value	Result
**2009**	0.4338	-0.0169	0.004916	6.4289	<0.01	Cluster
**2010**	0.4312	-0.0169	0.004920	6.3887	<0.01	Cluster
**2011**	0.4427	-0.0169	0.004947	6.5350	<0.01	Cluster
**2012**	0.4393	-0.0169	0.004942	6.4897	<0.01	Cluster
**2013**	0.4457	-0.0169	0.004985	6.5531	<0.01	Cluster

**Fig 4:**
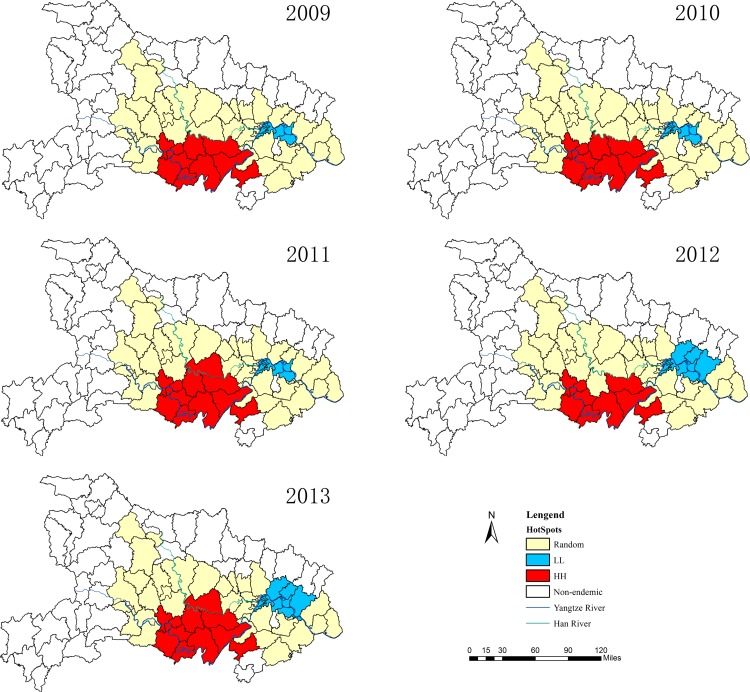
The local spatial autocorrelation distribution of human *S*. *japonicum* infection in Hubei Province from 2009 to 2013. (HH) High-high; (LL) Low-low.

The spatial clusters (hotspots) obtained from the local Gi* statistics for human *S*. *japonicum* infection in Hubei Province from 2009 to 2013 are shown in Fig. 4. The Z-score outcomes calculated by the Gi* statistics are categorized as clusters (hotspot or cold spot) or random at 5% significance level. During the five-year duration, the hotspots ranged from 9 to 11 counties (covering 12.70%–17.46% of the endemic counties (n = 63)) and the cold spots ranged from 6 to 12 counties (covering 9.52%–19.05% of the endemic counties (n = 63)) (Table 4). The hotspots are mainly distributed in the Jianghan Plain along the middle of the Yangtze River from 2009 to 2013, including the counties and cities of Jingzhou, Shashi, Jiangling, Gong’an, Shishou, Jianli, Honghu, and Chibi.

**Table 4:** The local autocorrelation analysis (Gi*) of *S*. *japonicum* infection in humans*.

Year	Hot spots		Cold spots	
	No.	Percentage (%)	No.	Percentage (%)
**2009**	10	15.87	6	9.52
**2010**	10	15.87	6	9.52
**2011**	11	17.46	7	11.11
**2012**	9	14.29	12	19.05
**2013**	11	17.46	11	17.46

*No.: number of counties that are hot spots or cold spots

## Discussion

The investigation of infectious disease spatial clustering has aroused more interest since the development of GIS and spatial statistics, which allow the quantification of the degree of clustering of infections [34–36]. GIS has been largely used to investigate the spatial epidemic characteristics of malaria [37,38], schistosomiasis [39–41], hemorrhagic fever with renal syndrome [36,42], trachoma [43], and so on. The transmission of *S*. *japonicum* is affected by many key factors, such as climatic suitability, spatial distribution of the intermediate host *O*. *hupensis*, and human activities [44,45]. Hence, *S*. *japonicum* infection exhibits marked spatial heterogeneity from the community scale to the regional scale, even at the single administrative village scale [46,47].

A study has reported that more than 80% of *S*. *japonicum* patients in China were distributed in the lake and marshland areas of Hubei, Hunan, Jiangxi, Anhui, and Jiangsu provinces [48]. In 2009, 136,142 cases of *S*. *japonicum* patients, 3,461 heads of infected bovines, and 76,667 hm^2^ of areas infested with *Oncomelania* snails in Hubei Province accounted for the highest numbers in China [49]. Therefore, an improved understanding of the spatial clustering of schistosomiasis in Hubei Province may provide useful insights in controlling the transmission of *S*. *japonicum* in the marshland regions of China.

Global and local cluster detection methods were used in this study to identify different types of clusters of schistosomiasis in Hubei Province. Moran’s *I* measures the spatial autocorrelation and evaluates the expressed pattern (clustered, dispersed, or random) based on both location and attribute information [33,50]. In general, a Moran’s *I* value near +1.0 indicates clustering, whereas a value near −1.0 indicates dispersion. The results of the global spatial autocorrelation analysis showed that Moran’s *I* statistics of *S*. *japonicum* infection were all above 0.4 (*P* < 0.01) during the five years, which indicated that *S*. *japonicum* had a positive spatial autocorrelation at the county level in Hubei Province from 2009 to 2013. The high prevalence areas were located along the Yangtze River from 2009 to 2013. This finding was similar to the spatial autocorrelation patterns in the Yangtze River basin of China [51]. Analogously, the spatial distribution of schistosomiasis was also nonrandom in the marshland areas of China at the county level [13]. The global spatial autocorrelation analysis results also showed that the spatial distribution of *S*. *japonicum* infections remained clustered and unchanged with time. Integrated control measures against schistosomiasis were carried out in Hubei Province [18], but the spatial distribution of *S*. *japonicum* infection did not obviously change over the course of control. This observation suggests that *S*. *japonicum* infection distributions may be mainly influenced by local natural and climatic conditions and by the geographic distribution of intermediate hosts (*O*. *hupensis*) and many vertebrate definitive hosts [52].

In this study, Gi* statistics was used to detect the local spatial autocorrelation of schistosomiasis in Hubei Province, and the hotspots are mainly located in the foci of Jianhan Plain along Yangtze River from 2009 to 2013. The results also suggested that the number of highly aggregated areas had little changed, ranging from 9 to 11 counties within the five-year analysis period. However, the number of less aggregated areas showed an increasing trend during the five years. This result may be because an integrated control strategy that emphasizes infection source control was carried in schistosomiasis-endemic areas of Hubei Province [18,19]. Hence, the prevalence of human *S*. *japonicum* infection declined, leading to an increase in the number of less aggregated areas. Meanwhile, the results suggested that the spatial distributions of high *S*. *japonicum* infections become less heterogeneous as the prevalence of infection decreases at the county level. Thus, the number of less aggregated areas increased in five years.

Global autocorrelation analysis identified the schistosomiasis-endemic clustering in Hubei Province on a global perspective, whereas local autocorrelation analysis further detected the locations with positive spatial clustering. Given that Hubei Province reached the schistosomiasis control criterion, *S*. *japonicum* transmission decreased and the accumulation scope changed. The spatial clustering proved to be significant to the distribution of human *S*. *japonicum* infection, and the high-cluster areas were mainly distributed in the Yangtze River Basin of Jianghan Plain area. These regions are the focus of future prevention and control. Furthermore, bovines contribute 80% or more to the local transmission in certain areas [53,54], and interventions targeting bovines can reduce the incidence of human infection [55].Thus, the integrated control strategy aimed at controlling the roles of humans and bovines as sources of *S*. *japonicum* infection should still be carried out.

Certain limitations in our research deserve further discussion. First, the spatial distribution of schistosomiasis in Hubei Province that were analyzed were at the county level, which may lead to a bias in the results. The spatial patterns of *Schistosoma* transmission are reportedly relevant to infection prevalence at a finer scale [39]. The infection prevalence analyzed in our study was at the county level, which was the average of the whole county. Furthermore, compared with smaller spatial scale level, the spatial distribution of *S*. *japonicum* infection may become less heterogeneous when analyzed at the county level. Further spatial autocorrelation analysis should be done to demonstrate the schistosomiasis spatial distribution at a much smaller spatial scale level, including town and village levels. Second, the diagnostic approach for schistosomiasis is not completely sensitive and specific. The miracidium hatching test is a traditional approach to assess *S*. *japonicum* infection, and its potential for high sensitivity has been recognized. However, even under optimal conditions, only 50% to 70% of eggs will hatch, with light infections being missed [22]. On the other hand, IHA has low specificity, just ranged from 85% to 90% [56]. Therefore, some precise methods for prevalence estimates need to be developed.

## Conclusions

In summary, our research highlighted the spatial epidemiological characteristics of schistosomiasis at the county level from 2009 to 2013 in Hubei Province, China. The results indicated that the hotspots of schistosomiasis are mainly located along Yangtze River, and the spatial distribution of *S*. *japonicum* infection did not obviously change over the course of control from 2009 to 2013. Our findings suggest that a spatial–temporal surveillance system should be established for identifying highly endemic regions and implementing timely prevention of schistosomiasis.

## Supporting Information

S1 File The prevalence of human *S*. *japonicum* infection of each county in Hubei Province from 2009 to 2013. (XLSX)
